# Imaging hydrogen peroxide in Alzheimer’s disease via cascade signal amplification

**DOI:** 10.1038/srep35613

**Published:** 2016-10-20

**Authors:** Jian Yang, Jing Yang, Steven H. Liang, Yungen Xu, Anna Moore, Chongzhao Ran

**Affiliations:** 1 Molecular Imaging Laboratory, Athinoula A. Martinos Center for Biomedical Imaging, Massachusetts General Hospital and Harvard Medical School, Boston, MA, 01890, USA; 2 School of Pharmacy, China Pharmaceutical University, Nanjing, 210009, China; 3 College of Pharmaceutical Sciences, Soochow University, Suzhou, 215006, China; 4 Division of Nuclear Medicine and Molecular Imaging & Center for Advanced Medical Imaging Sciences, Massachusetts General Hospital and Department of Radiology, Harvard Medical School, Boston, MA, 02114, USA.

## Abstract

In brains of Alzheimer’s disease (AD), reactive oxygen species (ROS) levels are significantly higher than that of healthy brains. Evidence suggests that, during AD onset and progression, a vicious cycle revolves around amyloid beta (Aβ) production, aggregation, plaque formation, microglia/immunological responses, inflammation, and ROS production. In this cycle, ROS species play a central role, and H_2_O_2_ is one of the most important ROS species. In this report, we have designed a fluorescent imaging probe CRANAD-88, which is capable of cascade amplifying near infrared fluorescence (NIRF) signals at three levels upon interacting with H_2_O_2_ in AD brains. We demonstrated that the amplification was feasible *in vitro* and *in vivo*. Remarkably, we showed that, for the first time, it was feasible to monitor the changes of H_2_O_2_ concentrations in AD brains before and after treatment with an H_2_O_2_ scavenger. Our method opens new revenues to investigate H_2_O_2_ in AD brains and can be very instructive for drug development.

Alzheimer’s disease (AD) is a terminal neurodegenerative disease, and no effective treatment is available so far. Based on the hallmarks of amyloid beta (Aβ) plaques and neurofibrillary Tau tangles, the so-called amyloid hypothesis and tau hypothesis have been proposed for AD pathology. However, the prevalent drug development strategies, which are primarily built on these hypotheses, have not been successful in delivering effective treatment^1^,^2^,^3^,^4^. This strongly indicates that other factors should also be taken into account in AD drug development. It has long been believed that AD is closely associated with oxidative stress, and evidence shows that in AD brains the reactive oxygen species (ROS) level is significantly higher than that in healthy control brains^5^,^6^,^7^. The increased ROS level can be attributed to multiple factors, such as over-accumulation of metal ions, aggregation of Aβs, inflammation, and microglia activation.

During the formation and growth of Aβ plaques, numerous ROS species are generated, and H_2_O_2_ can be considered to be one of the most important ROS species. H_2_O_2_ is produced through the Fenton reaction of Aβs with metal ions^8^,^9^,^10^,^11^. The generated ROS/H_2_O_2_ can further induce over-accumulation of inflammatory cytokines such as TNFα^12^,^13^, which can then attract microglia to encircle the active plaques^13^,^14^,^15^. The surrounding microglia release more ROS/H_2_O_2_, and contribute to the neuronal loss characteristic of this disease^15^. Studies show that interactions between microglia cells and the plaques can activate numerous receptors and enzymes. Among them, oxidative stress related NAD(P)H oxidase plays a crucial role^15^,^16^. Upon activation, NAD(P)H oxidase produces highly reactive ROS/H_2_O_2_^15^. This production of ROS/H_2_O_2_ further initiates production and crosslinking/aggregation of Aβs^17^,^18^,^19^, which in turn can lead to the generation of ROS/H_2_O_2_^8^,^9^,^10^,^11^. Moreover, recent data indicates that TNFα can also increase the production of Aβs^18^,^19^, which can additionally strengthen Aβ aggregation and crosslinking. According to the above facts, it is abundantly clear that a vicious cycle revolves around plaque formation, microglia/immunological responses, inflammation, ROS production, Aβ production and aggregation/crosslinking. As illustrated in Fig. 1a, ROS/H_2_O_2_ species play a central role. Therefore, detection of ROS/H_2_O_2_ in the AD brain *in vivo* is highly desirable. In this report, we have concentrated our efforts on the detection of H_2_O_2_ in AD brains.

Although H_2_O_2_ plays critical roles in numerous normal and abnormal biological processes, few references are available for the concentration of H_2_O_2_ in the brain of living beings, including human and mice, and it is not clear how the concentration of H_2_O_2_ changes under disease conditions such as AD^20^. Numerous imaging probes have been reported for detecting H_2_O_2_ in cells and in peripheral targets; however, none has been reported to detect H_2_O_2_ in brains under different disease status^21^,^22^,^23^,^24^,^25^,^26^,^27^. Clearly, it is an urgent need to provide an *in vivo* imaging tool to detect the H_2_O_2_ concentration in the living brain, particularly AD brains.

Several strategies for detecting H_2_O_2_ have been developed, and Chang’s boronate method and Nagano’s diketone method are the most validated approaches^25^,^26^,^27^. Although the reported H_2_O_2_ imaging probes are great for peripheral application and cell imaging^25^,^26^,^27^, the visualization of H_2_O_2_ in the brain is not very straightforward. Therefore it is still challenging to use them for *in vivo* imaging of H_2_O_2_ in AD brains.

In this report, we designed a curcumin analogue CRANAD-88, which has a capacity of three levels of cascade signal amplification. Remarkably, we demonstrated for the first time, that the changes of H_2_O_2_ concentrations from AD brains could be monitored before and after H_2_O_2_ scavenging. We believe that our method will be a valuable tool for investigating H_2_O_2_ in AD brains, and for AD drug development and treatment regime design.

## Results and Discussion

### Design of fluorescence imaging probe CRANAD-88

**Figure 1:**
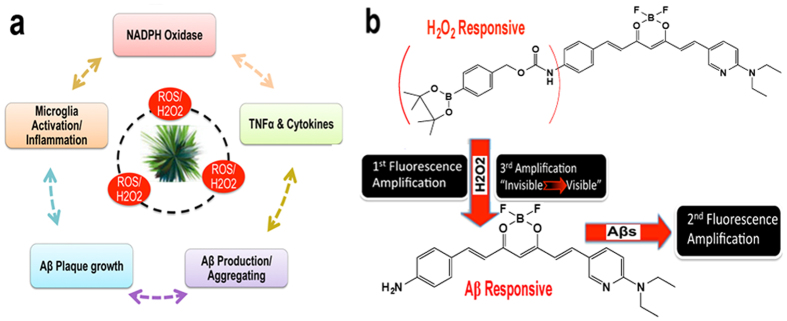
(**a**) The vicious cycle revolves around Aβ species and plaques; (**b**) Design of CRANAD-88 and the proposed three levels of cascade signal amplification.

Other researchers have reported various strategies to design H_2_O_2_ responsive probes^25^,^26^,^27^. Among them, boronate oxidation is one of the most validated approaches for the design of imaging probes that can increase fluorescent intensity after reaction with H_2_O_2_^25^,^26^. However, utilizing this method for cascade signal amplification has not been explored. In our previous studies, we demonstrated that CRANAD-58 was a “smart” NIRF probe for Aβs, and displayed about a 100-fold intensity increase after binding to Aβs^28^,^29^,^30^,^31^. To achieve cascade signal amplification, we integrated our previous Aβ “smart” probe strategy into the design of an H_2_O_2_ responsive probe. Through the integration, CRANAD-88 was designed by linking a close mimic of CRANAD-58 (Aβ responsive moiety) to a boronate (H_2_O_2_ responsive moiety) via a carbamate moiety (Fig. 1b). The benefit of the carbamate linker is that it leads the designed CRANAD-88 to be “invisible” in the near infrared spectrum. This invisibility is due to the carbamate’s electron-withdrawing effect, which shortens the probe’s excitation/emission wavelength. However, it becomes “visible” once it reacts with H_2_O_2_, because the boronate and carbamate moieties are removed to generate an electron-rich amino group for red-shifting. The probe’s reaction with H_2_O_2_, and the subsequent binding of the reaction intermediate with Aβs, as well as the red-shift all contribute to the cascade amplification of the NIRF signal (Fig. 1b).

### Synthesis and spectral characterization

**Figure 2:**
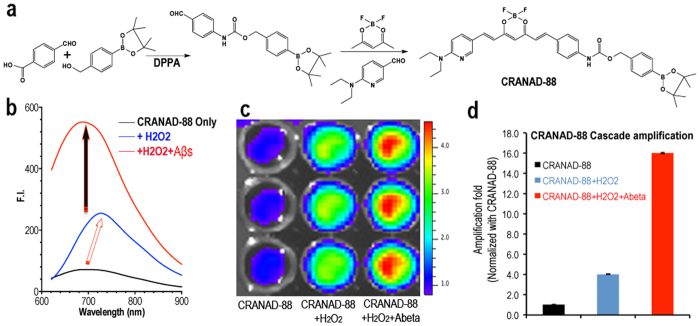
(**a**) The synthetic route for CRANAD-88; (**b**) Fluorescence intensity changes of CRANAD-88 (black line) with H_2_O_2_ (blue line, 1^st^ amplification and red-shift), and with H_2_O_2_ + Aβ aggregates (red line, 2^nd^ amplification). (**c**) Cascade signal amplification validation of CRANAD-88 with imaging parameters (Ex = 640 nm, Em = 700 nm) on a plate that was loaded with CRANAD-88 only (column 1), CRANAD-88 with H_2_O_2_ (column 2), and CRANAD-88 with H_2_O_2_ and Aβs (column 3), and (**d**) quantitative analysis of the image in (**c**).

As shown in Fig. 2a, we first prepared the boronate-attached benzaldehyde via Curtius reaction with DPPA (Diphenylphosphoryl azide) in a high yield (67%). CRANAD-88 was obtained based on our previous published procedures^28^,^29^,^30^,^31^. The excitation spectrum showed that its excitation peak is around 580 nm and emission was about 690 nm in PBS buffer with 20% DMSO (SI Fig. 1).

### *In vitro* solution testing for validating cascade fluorescence amplification

The emission peak of CRANAD-88 is around 690 nm, but its excitation peak is around 580 nm, therefore it is “invisible” for NIRF imaging (Ex > 640 nm required). To investigate whether CRANAD-88 is H_2_O_2_-responsive, we incubated CRANAD-88 in PBS buffer with various concentrations of H_2_O_2_. We found that the fluorescence intensity of CRANAD-88 was increased 3-fold after incubation with H_2_O_2_ (100 μM) at 37 °C (the first signal amplification) (Fig. 2b). We also observed significant red-shifts for both excitation and emission (Ex = 630 nm, Em = 730 nm) after the incubation (Fig. 2b). These red-shifts are very important for *in vivo* imaging, since a longer emission will allow imaging at greater depths and reduce background signal. Furthermore, these red-shifts convert “invisible” CRANAD-88 into a “visible” intermediate, which allows the probe to be visualized *in vivo* with NIRF imaging (the indirect signal amplification). To investigate whether the generated intermediate from the above incubation was responsive towards Aβs, we incubated the above solution with Aβ40 aggregates. As we expected, the fluorescence intensity of the intermediate was dramatically increased (2.5-fold) upon exposure to the aggregates (the second signal amplification) (Fig. 2b). To investigate whether the amplifications could be observed with parameters for *in vivo* imaging (Ex = 640 nm, Em = 700 nm), we conducted imaging (Ex = 640 nm, Em = 700 nm) with a plate that contained 1) CRANAD-88 only, 2) CRANAD-88 with H_2_O_2_, and 3) CRANAD-88 with H_2_O_2_ and Aβ40 aggregates. Consistent with solution spectral tests, significant increases could be observed from wells containing H_2_O_2_ (4-fold), and H_2_O_2_ with Aβs (16-fold) (Fig. 2c,d). Remarkably, amplifications from the plate imaging were significantly higher than that from solution spectra at 700 nm, due to the “invisibility” of CRANAD-88 with the imaging parameters. Our *in vitro* data suggested that three levels of cascade fluorescence signal amplification were attainable through H_2_O_2_-reponsive interactions, Aβ-responsive interactions, and transformation of the probe from “invisible” to “visible” in the near infrared spectrum.

Consistent with other reported boronate imaging probes^25^,^26^, CRANAD-88 showed higher H_2_O_2_ selectivity over other ROS species that include NO•, O_2_, ClO^−^ (SI Fig. 2a). It is also very sensitive to H_2_O_2_, a significant increase could be seen with 10.0 μM H_2_O_2_ (SI Fig. 2b). In addition, CRANAD-88 exhibited quick responses towards H_2_O_2_, and a significant increase was observed only after 10 minutes of incubation (SI Fig. 2c).

### Fluorescence responses of CRANAD-88 with Aβs without H_2_O_2_

When we incubated CRANAD-88 with various Aβs, but without H_2_O_2,_ increases in fluorescence intensity were observed. However, we also observed significant blue-shifts to 640 nm (SI Fig. 3). The data indicates that, without H_2_O_2_, CRANAD-88 was “invisible” in the presence of Aβs under conventional NIRF imaging parameters (Ex > 640 nm and Em > 650 nm required), due to its short excitation and emission.

### Phantom imaging with mouse brain homogenate

**Figure 3:**
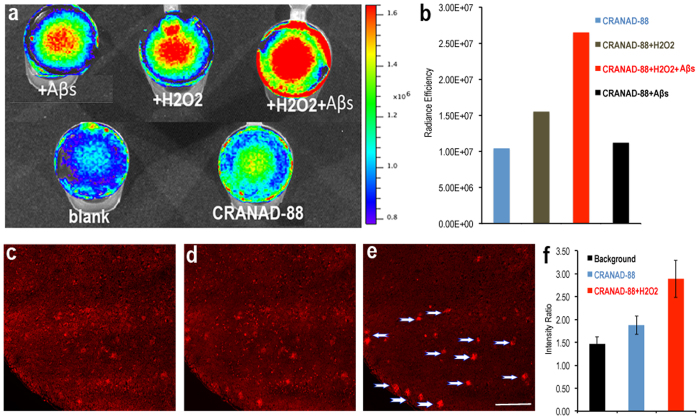
(**a**) Phantom imaging with brain homogenate. CRANAD-88 with Aβ 40 aggregates, with H_2_O_2_, with H_2_O_2_ + Aβ40 aggregates (upper panel), and brain homogenate blank, and CRANAD 88 only (low panel). (**b**) Quantification of (**a**) by normalizing with the signal from the blank brain homogenate. (**c**–**f**) Brain slice staining test with CRANAD-88. (**c**) Background of a brain slice; (**d**) Treated with CRANAD-88; (**e**) Treated with CRANAD-88 and H_2_O_2_ (100 μM); Plaques are indicated by white arrows. Scale Bar: 200 μm. (**f**) Quantitative analysis of intensity ratio of a plaque and vicinity background.

To validate whether CRANAD-88 can be used in a biologically relevant environment, we used brain homogenates from a wild type mouse to investigate the changes of CRANAD-88 after exposure to H_2_O_2_, Aβs, and both H_2_O_2_ and Aβs. We used an IVIS imaging system to collect data (Ex = 640 nm, Em = 700 nm). We observed an apparent signal increase after 30 minutes of incubation with H_2_O_2_ (500 nM), but no significant signal increase was observed after incubation with Aβs alone (Fig. 3a,b). This was because the probe was “invisible” with the imaging parameters until it was “turned on” by reaction with H_2_O_2_ (Fig. 3a,b). To confirm the cascade signal amplification in the homogenate, we further incubated the H_2_O_2_ containing homogenate with Aβ40 aggregates, and another significant increase of intensity could be observed (Fig. 3a,b). Taken together, the data suggested that the cascade signal amplification could be achieved in a biologically relevant environment.

### *In vitro* mouse brain slice microscopic imaging

To investigate whether we can observe the fluorescence intensity changes of CRANAD-88 in AD mouse brain slices, we first incubated the probe with a brain slice of an 11-month old APP/PS1 mouse for 30 minutes. There was no significant intensity increase compared to the slice before probe treatment. However, we found that several plaques lit up once the same brain slice was incubated with H_2_O_2_ at 37 °C for 30 minutes (Fig. 3c,d), and the lit-up plaques were well co-localized with thioflavin S staining (SI Fig. 4a,b). We quantified the signal using the fluorescence intensity ratio of the plaque of interest vs. an ROI of the same size of the vicinity of the plaque. We found that the amplification of the signal was about 2-fold with Aβ plaques and H_2_O_2_ when compared with the background of pre-probe treatment or CRANAD-88 only (Fig. 3f). These results strongly indicated that signal amplification could be observed in an AD brain slice, and is also Aβ specific.

### BBB penetrating studies

To investigate whether CRANAD-88 can cross BBB, we injected the probe into a wild type mouse, which was sacrificed and perfused at 30 minutes after the injection. The dissected brain was homogenized and extracted with ethyl acetate. Fluorescent spectral testing and LC-MS data from the brain extraction confirmed that CRANAD-88 was able to cross the BBB (SI Fig. 4c–e).

### *In vivo* imaging with CRANAD-88

**Figure 4:**
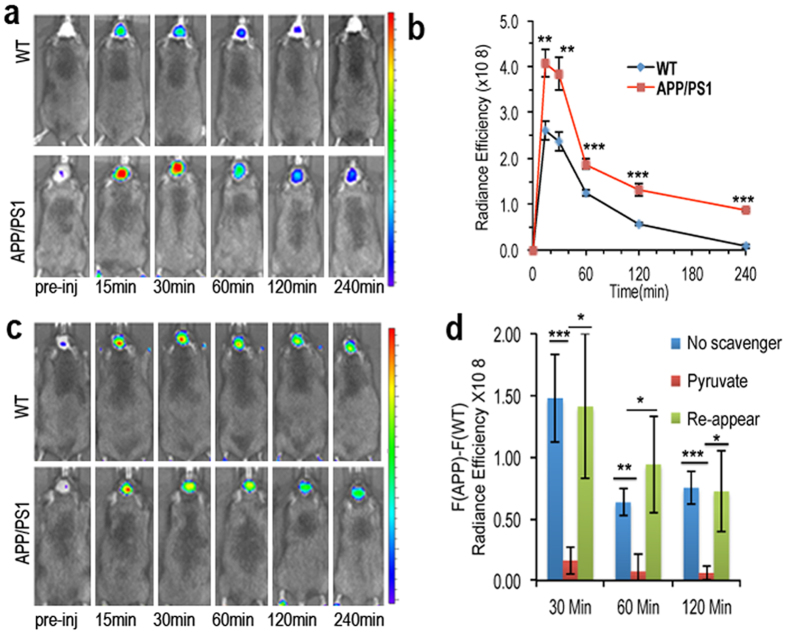
*In vivo* imaging study with CRANAD-88. (**a**) Representative images of WT and APP mice (0.1 mg/mice) at pre-injection, 15-, 30-, 60-, 120- and 240 min after injection. (**b**) The time course of CRANAD-88 with APP and WT mice. (**c**) Representative images of WT and APP mice, which both were treated with sodium pyruvate, d) the NIRF signal differences of CRANAD-88 between AD and WT mice (F_(APP)_ − F_(WT)_) treated without (blue bar) and with sodium pyruvate, and re-appearance of H_2_O_2_ one month after pyruvate scavenging (green bar). P value: *<0.05, **<0.01, and ***<0.005.

Few references are available for the concentration of H_2_O_2_ in the brain of living beings. It is not clear how the concentration of H_2_O_2_ changes under disease conditions such as AD. Studies suggest that the concentrations of H_2_O_2_ in AD brains are higher than that of normal brains^5^,^6^,^7^. However, direct evidence is still lacking. To prove the existence of elevated H_2_O_2_ in the AD mouse brain, we used CRANAD-88 to conduct NIRF imaging with transgenic AD model APP/PS1 mice, a well-studied transgenic model for AD research^32^,^33^. Age-matched wild type mice were used as the control group. We monitored the NIRF signal at 15-, 30-, 60-, 120-, and 240-minutes post injection. We found that the APP/PS1 group showed significantly higher fluorescence signal at all of the time points (1.56-, 1.62-, 1.51-, 2.32-, and 10.91-folds) than WT mice. This data indicated that the difference between APP/PS1 and WT reflected by CRANAD-88 was likely due to the cascade NIRF signal amplification (Fig. 4a,b).

### Monitoring H_2_O_2_ scavenging *in vivo*

To further confirm whether the above amplification is due to the existence of H_2_O_2_ in the brain, we used sodium pyruvate, which is a widely used H_2_O_2_ scavenger for cell culture^34^, and is capable of penetrating the BBB^35^. To conduct the experiments, we injected a mixture of CRANAD-88 and sodium pyruvate intravenously. As expected, the difference between the signals from the APP/PS1 group and the control group is significantly decreased (Fig. 4c,d, red bar), strongly indicating that amplification *in vivo* is due to H_2_O_2_ triggering and engagement with Aβs. In order to further investigate whether H_2_O_2_ would be re-produced after the clearance of sodium pyruvate, we imaged the same groups of mice after one month. We found that the difference between APP/PS1 and WT was restored (Fig. 4d, green bar), suggesting that H_2_O_2_ can be re-produced if toxic Aβ species are still existing.

Longitudinally monitoring the changes of H_2_O_2_ concentration could be very important for investigating the effects of H_2_O_2_ on the progression of AD pathology. From the design of CRANAD-88, it is clear that the total NIRF signal originates from the interaction with H_2_O_2_ and Aβs, and it is known that both Aβ and H_2_O_2_ concentrations change with ageing; therefore, the NIRF signal changes from CRANAD-88 can’t be used to quantitatively reflect the concentration change of H_2_O_2_ in the longitudinal studies. To quantitatively calculate the relative H_2_O_2_ changes during ageing and longitudinal therapy monitoring, we will use the ratio between CRANAD-88 and CRANAD-3 (R_H2O2_ = R_(CRANAD-88)_/R_(CRANAD-3)_). For CRANAD-3, our previous data showed that it could reflect the changes in Aβs during ageing and treatment^31^. Using the ratio of the two probes, we will be able to reliably calculate relative H_2_O_2_ changes in the longitudinal studies.

## Summary

H_2_O_2_ plays important roles for AD pathology. However, no method is available to detect the changes of H_2_O_2_ in the brain. In this report, results from *in vitro* spectral studies, plate imaging, phantom imaging, and brain slice imaging consistently suggested that three levels of cascade signal amplification was feasible. Remarkably, for the first time, we demonstrated that the changes of H_2_O_2_ concentrations in AD brain could be monitored with a NIRF imaging probe capable of cascade signal amplification. Our study is not only crucial for the proof of the existence of H_2_O_2_, but also points out several important questions that are needed to be addressed in terms of seeking effective treatments for AD. Our study strongly indicated that H_2_O_2_ can be reproduced if the Aβ plaques or other Aβs are still existing, and this pointed out that AD treatment is not only related to the removal of Aβs, but also related to ROS. Our study also pointed out another important question: whether Aβ-lowering treatment can lead to the decrease of ROS such as H_2_O_2_. If the Aβ-removal cannot reduce the ROS level, the toxic ROS will continuously damage the neurons. Therefore, ROS reduction should be considered to be one type of treatment outcome to be evaluated. In summary, our method can be used to monitor the changes of H_2_O_2_ in brains of AD mice, and this technology will be an important tool for AD drug development and treatment regime management.

## Materials and Methods

Reagents used for the synthesis were purchased from Sigma-Aldrich and used without further purification. The pH of the PBS buffer was 7.4. Column chromatography was performed on silica gel (SiliCycle Inc., 60 Å, 40–63 mm) slurry packed into glass columns. Synthetic Aβ peptides (1–40/42) were purchased from rPeptide (Bogart, GA, 30622). Aggregates for *in vitro* studies were generated by the slow stirring of Aβ40 in PBS buffer for 3 days at room temperature. CRANAD-88 was dissolved in DMSO to prepare a 25.0 μM stock solution. ^1^H and ^13^C NMR spectra were recorded at 300 and 125 MHz, respectively, and reported in ppm downfield from tetramethylsilane. Fluorescence measurements were carried out using an F-4500 fluorescence spectrophotometer (Hitachi). Transgenic female APP-PS1 mice and age matched wild-type female mice were purchased from Jackson Laboratory. All animal experimental procedures were approved by the Institutional Animal Care and Use Committee (IACUC) at Massachusetts General Hospital, and carried out in accordance with the approved guidelines.

### Synthesis of CRANAD-88

#### 4-(4,4,5,5-Tetramethyl-1,3,2-dioxaborolan-2-yl) benzyl 4-formyl-phenylcarbamate

4-Formylbenzoic acid (1.0 g, 6.7 mmol) was dissolved in 1,4-dioxane (14 mL), followed by the addition of triethylamine (0.93 mL, 6.7 mmol), DPPA (1.6 mL, 7.3 mmol), and 4-(hydroxymethyl) phenylboronic pinacol ester (1.7 g, 7.3 mmol). The resulting mixture was heated to 100 °C for 2 hours. After evaporating off the solvent, the residue was purified with a silica gel flash column chromatography (Hexanes/ethyl acetate = 3/2) to afford the intermediate as a pale yellow solid (1.7 g, yield: 66.7%).

^1^H NMR (CDCl_3_): δ(ppm) 1.34 (s, 12H), 5.23 (s, 2H), 7.06 (br, s, 1H), 7.37 (d, 2H, *J* = 7.8 Hz), 7.54 (d, 2H, *J* = 8.4 Hz), 7.81–7.84 (m, 4H), 9.89 (s, 1H). ^13^C NMR (CDCl_3_): δ(ppm) 191.9, 152.6, 143.4, 138.4, 135.1, 135.0, 131.7, 131.2, 127.4, 118.0, 83.9, 67.3, 24.8 ESI-MS (M + H) m/z = 382.1.

#### CRANAD-88

To a solution of the above intermediate (209 mg, 0.54 mmol) in acetonitrile (3.0 mL), 2,2-Difluoro-1,3-dioxaboryl-pentadione crystals (60 mg, 0.54 mmol) was added, followed by the additions of acetic acid (80 μL), tetrahydroisoquinoline (16 μL), and 4-(dimethylamino)-benzaldehyde (71.3 mg, 0.54 mmol). The resulting solution was stirred at 60 °C overnight. A black residue was obtained after removing the solvent and further purified with a flash column (Hexane/Ethyl acetate = 1/1) to give a black power CRANAD-88 (8.0 mg, yield: 2.2%).

^1^H NMR (CDCl_3_) δ (ppm) 1.23–1.25 (t, 6H), 1.34 (s, 12H), 3.56–3.63 (q, 4H), 5.22(s, 2H), 5.95 (s, 1H), 6.41 (d, *J* = 15.3 Hz, 1H), 6.51 (d, *J* = 9.3 Hz, 2H), 6.56 (d, *J* = 15.6 Hz, 1H), 6.86 (s, 1H), 7.38 (d, *J* = 8.1 Hz, 2H), 7.44 (d, *J* = 8.4 Hz, 2H), 7.53 (d, *J* = 8.7 Hz, 2H), 7.67 (dd, *J*_*1*_ = 2.4 Hz, *J*_*2*_ = 9 Hz, 1H), 7.81 (d, *J* = 8.1 Hz, 2H), 7.88 (d, *J* = 15.6 Hz, 1H), 7.95 (d, *J* = 15.3 Hz, 1H), 8.35 (d, *J* = 2.1 Hz, 1H); ^13^C NMR (CDCl_3_) δ (ppm) 12.88, 24.81, 43.13, 67.2, 83.87, 101.46, 106.17, 114.48, 118.08, 118.54, 119.44, 127.35, 129.49, 130.10, 135.06, 135.73, 138.57, 140.67, 144.83, 145.96, 152.71, 153.49, 158.78, 177.5, 179.72; ^19^F NMR (CDCl_3_) δ (ppm) 141.44, 141.38; ESI-MS (M + H) m/z = 672.2.

#### Fluorescence spectral testing of CRANAD-88 with H_2_O_2_, Aβ aggregates and H_2_O_2_+ Aβ aggregates

To record the fluorescence response of CRANAD-88 with different solutions, we utilized the following procedures. Step 1: 1.0 mL of PBS buffer with 20% DMSO was added to a quartz cuvette as a blank control and its fluorescence was recorded with the same parameters as for CRANAD-88. Step 2: The fluorescence emission spectrum of a CRANAD-88 solution (1.0 mL, 250 nM) was recorded with excitation at 560 nm and emission from 580 to 900 nm. Step 3: To the above incubated CRANAD-88 solution, 10 μL H_2_O_2_ was added (final concentration 100 μM). The emission spectra at different time points were recorded (Ex = 560 nm, Em = 580–900 nm). Step 4: To the above solution of CRANAD-88 and H_2_O_2_, 10 μL of Aβ 40 aggregates were added to make the final Aβ concentration of 250 nM. The emission spectra of the resulting mixture at different time points were recorded as described in Step 2. The final spectra from steps 2 and 3 were corrected using the blank control from Step 1.

#### BBB penetration of CRANAD-88

Similar procedure from ref. 30 was followed. The ethyl acetate extraction was subjected to fluorescence spectra recording and LC-MS analysis.

#### Brain phantom studies

A 4-month old balb/c mouse was perfused and sacrificed, and its brain was dissected. The brain was homogenized with 2.0 ml PBS, and the homogenate was equally divided into five eppendorf tubes and diluted to 1 ml with PBS. Every tube had added 20 μL CRANAD-88 (final concentration 500 nM) except the control of homogenate blank. No. 1: CRANAD-88 only; No. 2: CRANAD-88 and 20 μL H_2_O_2_ (final concentration 10 μM); No. 3: CRANAD-88 and H_2_O_2_ (final concentration 10 μM) incubated for 30 minutes at 37 °C, and then 20 μL Aβ40 aggregates (final concentration 500 nM) was added; No. 4: CRANAD-88 and 20 μL Aβ40 aggregates (final concentration 500 nM). The tubes were imaged with EX/EM = 640/700 nm on an IVIS Spectrum imaging system.

#### Brain slice testing

A brain slice (25 micron) of an 11-month old APP/PS1 mouse was washed with double-distilled water for 5 minutes, then fixed with 4% formalin for 5 minutes, and washed with double-distilled water. First, a background image of a brain slice was taken using a fluorescence microscope before probe treatment. Second, an image was taken after the slice was incubated with CRANAD-88 (25 μM) for 30 minutes. Third, an image was taken after the above slice was incubated with H_2_O_2_ (100 μM) at 37 °C for 30 minutes.

#### In Vivo NIRF Imaging

*In vivo* NIRF imaging was performed using an IVIS Spectrum animal imaging system (Caliper LifeSciences, Perkin-Elmer, Hopkinton, MA). Images were acquired with a 640 nm excitation filter and a 700 nm emission filter. Data analysis was performed using Living Image 4.2.1 software. 15-month old transgenic APP-PS1 mice (female, n = 3–4) and age-matched wild-type control mice (female, n = 3–4) were shaved to remove the fur from the brain area before background imaging. An injection solution of CRANAD-88 (4.0 mg/kg) was freshly prepared in 15% DMSO, 15% cremorphor, and 70% PBS, and the solution was stabilized for 20 min before injection. Each mouse was intravenously injected with 100 μL of CRANAD-88 via tail vein. Fluorescence signals from the brain areas were recorded at 0-, 15-, 30-, 60-, 120-, and 240-min after the intravenous injection. To quantify the NIRF signal, an equal size ROI was drawn around each brain region.

#### Monitoring H_2_O_2_ scavenging in vivo

The same mice from the above experiments were used after waiting for one week. *In vivo* imaging data indicated that the injected CRANAD-88 was totally washed out. For scavenging H_2_O_2_, 100 mM sodium pyruvate solution (sigma) was used. Both WT and APP/PS1 mice were i.v. injected with 100 μL of CRANAD-88 (same dose and formulation as the above experiments) and 100 μL of sodium pyruvate at the same time. After injection, the mice were imaged using the same imaging protocol as the above experiments.

## Additional Information

**How to cite this article**: Yang, J. *et al.* Imaging hydrogen peroxide in Alzheimer’s disease via cascade signal amplification. *Sci. Rep.* **6**, 35613; doi: 10.1038/srep35613 (2016).

## Supplementary Material

Supplementary Information
