# Supplementary material for: Imaging hydrogen peroxide in Alzheimer’s disease via cascade signal amplification
Source: Sci Rep. 2016 Oct 20;6:35613. doi: 10.1038/srep35613 (PMC5071891; doi:10.1038/srep35613)
Supplement: Supplementary Information [file srep35613-s1.pdf]

Imaging hydrogen peroxide in Alzheimer's disease via cascade signal amplification

Jian Yang[1,2], Jing Yang[1,3], Steven H. Liang[4], Yungen Xu[2*], Anna Moore[1], and Chongzhao Ran[1]*

[1]Molecular Imaging Laboratory, Athinoula A. Martinos Center for Biomedical Imaging, Massachusetts General Hospital and Harvard Medical School, Boston, MA, 01890;

[2]School of Pharmacy, China Pharmaceutical University, Nanjing, China, 210009;

[3]School of Pharmacy, Soochow University, Suzhou, China, 215006;

[4]Division of Nuclear Medicine and Molecular Imaging & Center for Advanced Medical Imaging Sciences, Massachusetts General Hospital and Department of Radiology, Harvard Medical School, Boston, MA, 02114.

Corresponding authors: Chongzhao Ran, cran@nmr.mgh.harvard.edu
Yungen Xu, xyg64@126.com

Supplemental Figures

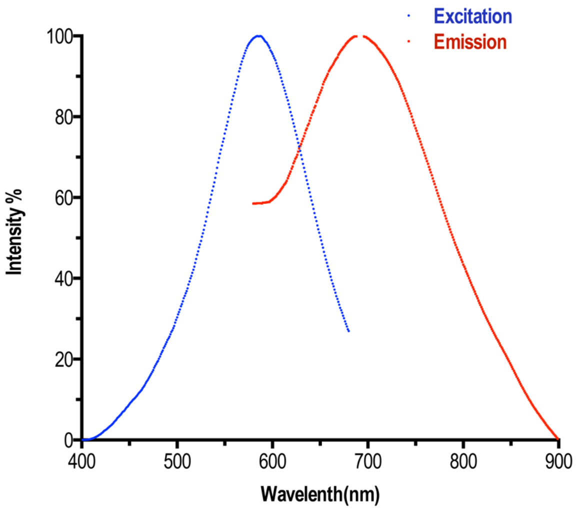

SI Fig.1 The excitation and emission spectra of CRANAD-88 in 20%DMSO PBS buffer.

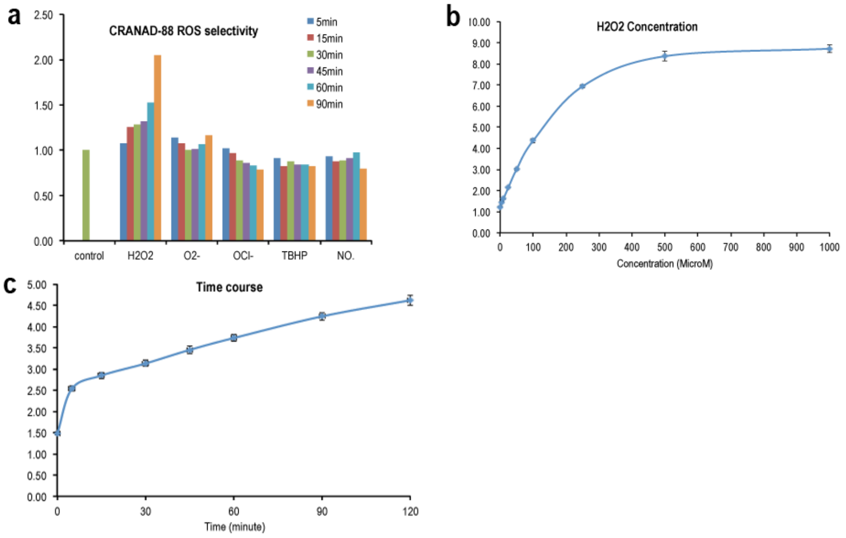

SI Fig.2 a) The selectivity of CRANAD-88 for H_2O_2 over other ROS species; b) The titration curve of CRANAD-88 with different concentrations of H_2O_2; c) Time course of CRANAD-88 with H_2O_2 (100μM stock solution);

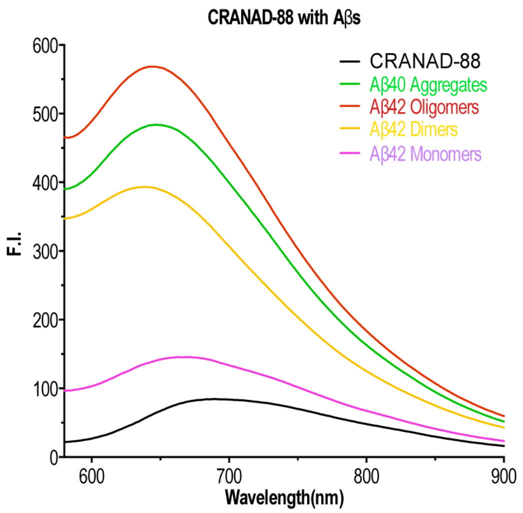

SI Fig.3 The emission spectra of CRANAD-88 with various Aβ species.

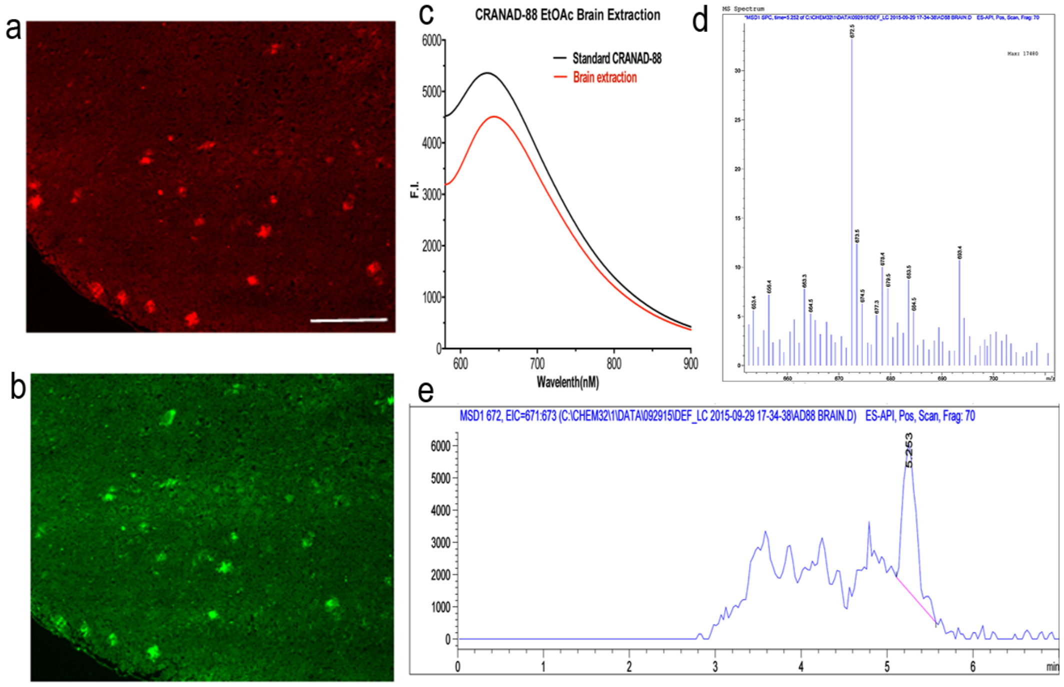

SI Fig.4 Co-staining with Thioflavin S and BBB penetration investigation of CRANAD-88. a-b) Co-staining of a brain slice of an APP/PS1 mouse with thioflavin S (green) and CRANAD-88/H_2O_2 (red). c) Fluorescence spectra of CRANAD-88 standard in ethyl acetate, and brain ethyl acetate extraction after i.v. injection of CRANAD-88; d) Mass spectrum of CRANAD-88 from the brain extraction; e) ion extracted LC-MS of CRANAD-88 from the brain extraction.

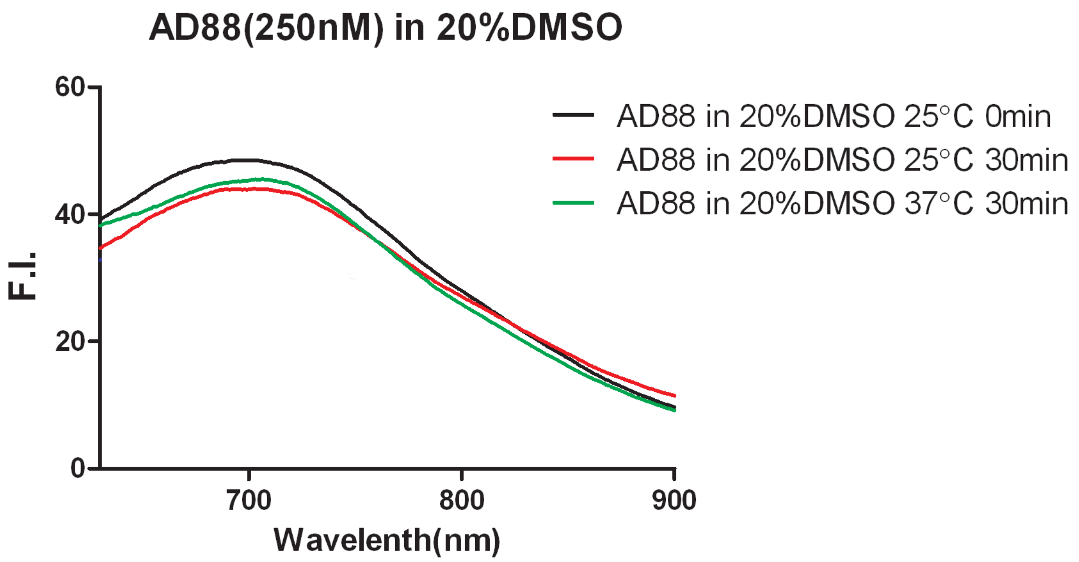

SI Fig.5 The stability of CRANAD-88 in 20% DMSO/PBS solution. There is no significant fluorescent intensity change after incubated at 37°C.
